# Release of Proteins from Intact Chloroplasts Induced by Reactive Oxygen Species during Biotic and Abiotic Stress

**DOI:** 10.1371/journal.pone.0067106

**Published:** 2013-06-14

**Authors:** Kwang-Chul Kwon, Dheeraj Verma, Shuangxia Jin, Nameirakpam D. Singh, Henry Daniell

**Affiliations:** 1 Department of Molecular Biology and Microbiology, College of Medicine, University of Central Florida, Orlando, Florida, United States of America; 2 Departments of Biochemistry and Pathology, University of Pennsylvania, School of Dental Medicine, Philadelphia, Pennsylvania, United States of America; Purdue University, United States of America

## Abstract

Plastids sustain life on this planet by providing food, feed, essential biomolecules and oxygen. Such diverse metabolic and biosynthetic functions require efficient communication between plastids and the nucleus. However, specific factors, especially large molecules, released from plastids that regulate nuclear genes have not yet been fully elucidated. When tobacco and lettuce transplastomic plants expressing GFP within chloroplasts, were challenged with *Erwinia carotovora* (biotic stress) or paraquat (abiotic stress), GFP was released into the cytoplasm. During this process GFP moves gradually towards the envelope, creating a central red zone of chlorophyll fluorescence. GFP was then gradually released from intact chloroplasts into the cytoplasm with an intact vacuole and no other visible cellular damage. Different stages of GFP release were observed inside the same cell with a few chloroplasts completely releasing GFP with detection of only red chlorophyll fluorescence or with no reduction in GFP fluorescence or transitional steps between these two phases. Time lapse imaging by confocal microscopy clearly identified sequence of these events. Intactness of chloroplasts during this process was evident from chlorophyll fluorescence emanated from thylakoid membranes and in vivo Chla fluorescence measurements (maximum quantum yield of photosystem II) made before or after infection with pathogens to evaluate their photosynthetic competence. Hydrogen peroxide and superoxide anion serve as signal molecules for generation of reactive oxygen species and Tiron, scavenger of superoxide anion, blocked release of GFP from chloroplasts. Significant increase in ion leakage in the presence of paraquat and light suggests changes in the chloroplast envelope to facilitate protein release. Release of GFP-RC101 (an antimicrobial peptide), which was triggered by Erwinia infection, ceased after conferring protection, further confirming this export phenomenon. These results suggest a novel signaling mechanism, especially for participation of chloroplast proteins (e.g. transcription factors) in retrograde signaling, thereby offering new opportunities to regulate pathways outside chloroplasts.

## Introduction

Chloroplasts support life on earth by performing photosynthesis. In addition to carbohydrates, plastids synthesize amino acids, proteins, fatty acids, pigments, hormones, vitamins and therapeutic biomolecules. Because <100 proteins are synthesized via the plastid genome, several thousand proteins are imported from the cytoplasm to carry out these diverse metabolic and biosynthetic functions. Therefore, coordination and assembly of multi-subunit complexes or biosynthetic pathways encoded by the plastid and nuclear genome requires efficient and accurate signaling between these two cellular compartments. The anterograde signaling pathways, in which the nucleus encodes plastid protein subunits, transcription factors and RNA binding proteins to coordinate plastid functions [1], have been studied in depth. Although it has been known for several decades that nuclear gene expression is also regulated by plastids via retrograde signaling, the molecular mechanism is still unknown [2]. Biochemical and genetic approaches so far have not been successful but a systems biology approach might provide new insights into retrograde signaling pathways [1]. Early studies indicated that Mg-protoporphyrin IX (Mg-Proto IX), an intermediate of tetrapyrrole pathway, served as a retrograde signaling molecule [3]. However, recent studies found inadequate connection of Mg-Proto IX steady state levels with transcription of nuclear deciphered genes [4], [5]. Further studies have not yet identified tetrapyrrole intermediates as absolute messengers or the mechanistic aspects of their involvement in activation of signal transduction pathways from plastids to the nucleus [2]. It has been shown that membrane-bound transcription factors (MTFs) regulate various cellular functions through a proteolytic activation mechanism [6]–[8]. Recently PTM, a chloroplast envelope-bound plant homeodomain transcription factor, has been shown to be involved in retrograde signal pathways [9]. It is likely that retrograde signals from plastids during development (greening) are different from those generated under stress and might involve transcripts, proteins or other catalytic biomolecules.

Although a plethora of proteins are imported into plastids in a unidirectional manner [10], there is no report of export of any protein synthesized within plastids. Different pathways for protein targeting of nuclear-encoded genes into the chloroplast have been examined [11]. Proteins augmenting the chloroplast import pathways have also been investigated [12]. However, several recent studies indicate that proteins and/or transcripts could be exported from plastids. For example, when Tic40, a protein within the import complex localized in the inner plastid envelope was expressed within chloroplasts via the chloroplast genome, all other inner membrane proteins encoded by the nuclear genome were highly upregulated [13], suggesting retrograde signal transduction initiated by Tic40, in healthy plants. When antimicrobial peptides were expressed through the chloroplast genome, they conferred protection against bacterial or viral pathogens [14], [15]. Lysis of plastids to release antimicrobial peptides could offer a simplistic explanation but retention of antimicrobial peptides within plastids did not support this hypothesis [15]. Furthermore, expression of biomass hydrolyzing enzymes within chloroplasts, again conferred very high levels of protection to plant pathogens [16], indicating a hypersensitive response triggered by proteins expressed within plastids. In the course of tobacco mosaic virus (TMV) infection, a chloroplast localized receptor interacting protein (NRIP1) showed interaction with the P50 helicase in the cytoplasm [17]. In fact, this is not an exception since a large number of nucleotide-binding receptors are localized within the chloroplasts. The majority of *Pseudomonas syringae* secreted proteins have chloroplast targeting signal sequences, requiring retrograde signaling to the nucleus for eliciting the defense responses [18].

In order to investigate the anterograde/retrograde signaling between plastids and nucleus, and protein export, in this study we used green fluorescent protein (GFP) as a reporter expressed via the chloroplast genome in two unrelated species (tobacco and lettuce). The movement of GFP upon infection with *Erwinia carotovora* in both species was followed using time lapse confocal imaging. In parallel, we investigated movement of GFP under abiotic stress using paraquat and the signal transduction pathway under both biotic and abiotic stress.

## Materials and Methods

### Chloroplast transformation vectors

To construct vectors for chloroplast transformation, overlapping primers containing flanking restriction enzyme sites (Forward *Sal*I and Reverse *Nde*I) were designed for amplification of protein transduction domain (PTD, 16 amino acids – RHIKIWFQNRRMKWKK) of PDX-1 (pancreatic and duodenal homeobox factor-1) [19], fused to lettuce endogenous *psb*A 5′ untranslated region (LsPpsbA). PCR was carried out using overlapping primers and pDVI-1 vector [20] as the template. The PCR end product was resolved by electrophoresis in agarose gel and the fused DNA fragment comprised of LsPpsbA-PTD was extracted from gel followed by cloning into pCR BluntII Topo vector (Invitrogen). Soluble modified green fluorescent protein (GFP) coding sequence was PCR amplified using sequence defined primers with flanking restriction sites (Forward *Nde*I and Reverse *Xba*I) using pLD-GFP-His6-Factor Xa-retrocyclin-101 (RC101) vector [15] as the template and ligated to pCR BluntII Topo vector. The LsPpsbA-PTD and GFP sequence was confirmed by sequencing to make sure that no errors were introduced during PCR amplification. The LsPpsbA-PTD sequence was released from pCR BluntII Topo vector and ligated into the pDVI-1 vector resulting in pDVI-PTD. The GFP coding sequence was excised by partial digestion with *Nde*I and complete digestion with *Xba*I and ligated to pDVI-PTD. The GFP expression cassette was cloned into the pLsDV vector using *Sal*I and *Not*I restriction enzymes resulting in pLs-PTD-GFP vector for lettuce transformation. Also, the GFP expression cassette was cloned into the pLD vector utilizing *Sal*I and *Xba*I restriction enzymes resulting in pLD-PTD-GFP for tobacco chloroplast transformation. All cloning steps were completed in *Escherichia coli* following benchmark molecular biology procedures [21].

### Generation and molecular characterization of transplastomic plants

Fully expanded leaves of tobacco and lettuce were bombarded using the biolistic device PDS1000/He and transplastomic lines were recovered as explained previously [20], [22]. Molecular characterization was performed as described earlier [22], [23]. The Qiagen DNeasy plant mini kit was used to isolate genomic DNA from plant leaves. PCR assay was done to verify transgene integration within the inverted repeat region of the chloroplast genome, utilizing two primer sets 3P?3M and 5P?2M for tobacco or 16SF/3M and 5P/2M for lettuce respectively [24], [25]. The PCR was carried out as described before [22], [23]. Further rounds of selection were done to create homoplasmic lines as already described [22], [23]. Previously established lab protocol for Southern blot analysis was carried out to evaluate homoplasmy [22], [23]. Briefly, total plant genomic DNA (1–2 µg) extracted from untransformed and transplastomic plants were digested with *Sma*I for lettuce and *Hind*III for tobacco. The digested product was resolved in agarose gel and blotted onto nylon membrane. The flanking sequence (0.81 kb) comprising of the *trn*I/*trn*A genes was labeled with ^32^P [dCTP] and used as a probe for hybridization with nylon membrane using Stratagene QUICK-HYB hybridization solution following manufacturer's protocol. Southern positive plants were transferred to greenhouse.

Protein was extracted from PTD-GFP tobacco and lettuce transplastomic leaves as previously described [15]. The homogenized plant extract was collected and the total soluble protein (TSP) concentration for homogenate and supernatant was obtained by the Bradford assay. Different concentrations of TSP along with known quantity of GFP standards were resolved by 12% polyacrylamide gel electrophoresis. The GFP fusion proteins were examined in the resolved gel by AlphaImager^®^ and AlphaEase^®^ FC software (Alpha Innotech). The percent TSP of GFP fusion protein was calculated by comparing integrated density value of samples with known quantities of the GFP standards. Values are represented as means ± SD from three independent experiments.

### Confocal microscopic analysis of GFP movement under biotic or abiotic stress

To investigate movement of GFP under biotic stress tobacco and lettuce transplastomic leaves were inoculated with *E. carotovora* suspension culture. Transplastomic lines were also inoculated with the bacterial culture media without *E. carotovora* to serve as control. *E. carotovora* strain received from Dr. Jerry Bartz's laboratory (University of Florida, Gainesville) was cultured in nutrient broth (NB) medium for 24 hr at 25°C. Leaf discs were made using cork borer (9 mm in diameter), then infected with *E. carotovora* (OD_600_ = 0.2) in a multi-well culture plate for 1–2 hr duration. For control, leaf discs were inoculated with nutrient broth medium under the same condition. After incubation leaf discs were washed with distilled water and prepared for observation under confocal microscope. Leica TCS SP5 II confocal microscope was used for laser scanning. An argon laser at 488 nm wavelength was used to excite GFP and emission was recorded between 500 and 600 nm. Time lapse images were also captured using same Leica microscope after 30 min of Erwinia infection. For the investigation of GFP movement under abiotic stress, paraquat with or without Tiron was treated as described below. Each experiment was repeated five times and each time two leaves from at least two different lines were used to make six discs per treatment.

### Staining and quantification of ROS

To investigate abiotic stress, leaf discs were collected from at least five different plants, randomized and then preincubated in water for 2 hr under dim light. After preincubation, leaf discs were divided into groups of 20 for each experiment then soaked in Tween 20 (0.1 %) containing water with or without 16 μM paraquat (methyl viologen dichloride hydrate, Sigma) and vacuum infiltrated for 2 min. Visualization and measurement of superoxide and hydrogen peroxide were carried out as described earlier [26] with suitable modifications. Superoxide was detected with nitroblue tetrazolium (NBT) (Sigma). Leaf discs infected with *E. carotovora* and treated with paraquat were immersed in NBT-containing solution (1 mg/ml for *E. carotovora*, and 0.5 mg/ml for paraquat treatment) in 10 mM potassium phosphate buffer (pH 7.8) including 10 mM sodium azide and vacuum infiltrated for 2 min. For decolorization of chlorophyll, colored leaf discs were boiled in acetic acid-glycerol-ethanol (1/1/3) (v/v/v) solution at 95°C for 5 min. For photography, leaf discs were kept in glycerol-ethanol (1/4) (v/v) solution. For quantification of superoxide, formazan-precipitated blue leaf discs were ground in liquid nitrogen, then solubilized in 2 M KOH-DMSO (1/1.16) (v/v). After spin-down to remove debris, supernatant was measured at A_630_ and compared with a standard curve which was plotted with known amount of NBT in the KOH-DMSO mix. Hydrogen peroxide was visualized with 3,3′-diaminobenzidine (DAB) (Sigma) suspended in water (pH 3.8 with KOH). DAB solution was always made fresh in order to preclude oxidation. Leaf discs were submerged and vacuum infiltrated with DAB solution (1 mg/mL) for 10 min. For quantification of hydrogen peroxide, leaf discs were powdered in liquid N_2_ and homogenized in 0.2 M perchloric acid (HClO_4_) then spun down to remove debris. Supernatant was evaluated at A_450_ and quantified by comparing with a standard curve which was plotted with known concentrations of H_2_O_2_ in 0.2 M HClO_4_-DAB. The amount of H_2_O_2_ and formazan was plotted as mean ± SD from three independent experiments against time. Tiron (Acros Organics), inhibitor of superoxide anion, was treated as described previously [27]. For Tiron pretreatment, leaves were cut out at the base of petiole with a scalpel and the sliced petioles were soaked in water containing 0, 1 and 2.5 mM Tiron for 30 min under light (250 µmole/m^2^/sec). The leaves were further incubated for another 1 hr with paraquat (16 μM) containing Tiron or excluding Tiron.

### Measurements of ion leakage after paraquat treatment

Ion leakage from leaf discs was measured as described earlier [28]. Leaf discs were treated with paraquat (16 μM) under light as described above. After treatment, six leaf discs were floated on 8 ml of H_2_O for 12 hr at 4°C followed by measuring conductivity of bathing solution using a conductivity meter (Model 220, Denver Instrument). The data was recorded as value A. After that, the leaf discs were put back into the bathing solution and incubated for 30 min at 95°C. When the bathing solution was cooled down to room temperature, conductivity was measured once more and recorded as value B. Ion leakage of samples was presented as (value A/value B)×100 =  %. The percentage of ion leakage was represented as means ± SD of three independent experiments.

### *In planta* bioassays with *Erwinia carotovora*

*E. carotovora* suspensions containing 10^8^, 10^6^, 10^4^ and 10^2^ cells were made from overnight grown culture of *E. carotovora* and injected into leaves of PTD-GFP and GFP-RC101 (transplastomic tobacco plants expressing the antimicrobial peptide Retrocyclin-101 fused with GFP), using a syringe as described previously [15]. At the same time, 20 μl of double distilled water was injected into the PTD-GFP and GFP-RC101 tobacco leaves to serve as control. Leaves were photographed on 5 dpi (day post inoculation). Each experiment was repeated three times and each value denotes mean of triplicates with standard deviations. The colonization of *E. carotovora* in PTD-GFP and GFP-RC101 plants was investigated according to our previous report [14]. In brief, 20 μl of bacterial suspension (1.0×10^5^ cells) were injected into PTD-GFP and GFP-RC101 tobacco leaves through a syringe as described previously. Leaf discs were cut out from the inoculated areas of individual plants after 1, 2, 3 dpi (day post inoculation). The bacterial colonization in the leaf discs was measured as described previously [14]. Using the inoculation site as centre of a circle, the infiltrated area of individual plant was excised from the inoculated leaves for confocal microscope analysis on 24 and 48 hr after *E. carotovora* infection.

The plant cells were observed under confocal microscope (Leica) at 488 nm excitation wavelengths for GFP (green) and a 633 nm for chlorophyll autofluorescence (red). Images within the same section of a figure were obtained using identical confocal settings and adjusted equally. Each experiment was carried out at least three times with independent samples, and representative numbers are presented as means ± SD.

### Measurement of PSII quantum yield by Fv/Fm

PSII maximum efficiency was calculated using a portable chlorophyll fluorometer PAM-2100 (Walz) at room temperature. Leaves from wild type (untransformed), PTD-GFP and GFP-RC101 transplastomic plants were inoculated with *E. carotovora* (OD_600_ = 10^5^) using syringe and incubated for 24 and 48 hr at room temperature. Before measuring fluorescence emission, the leaves were incubated in the dark for >30 min. All measurements were performed as described previously [29]. The PSII quantum yield was computed from Chlorophyll a (Chla) fluorescence as *F*_v_′/*F*_m_′  =  (*F*_m_′− *F*)/*F*_m_′. More than ten measurements were made from two different plants for each plant type. Values are represented as means ± SD.

### Statistical evaluation

Pairwise statistical analysis for quantification of bacterial population after Erwinia infection was performed by one-way analysis of variance (single factor ANOVA). Differences with *P*<0.05 were deemed significant (*, *P*<0.05; **, *P*<0.001; ***, *P*<0.0001 vs control group). Values are represented as means ± SD.

## Results

### Generation and molecular characterization of transplastomic plants

**Figure 1:**
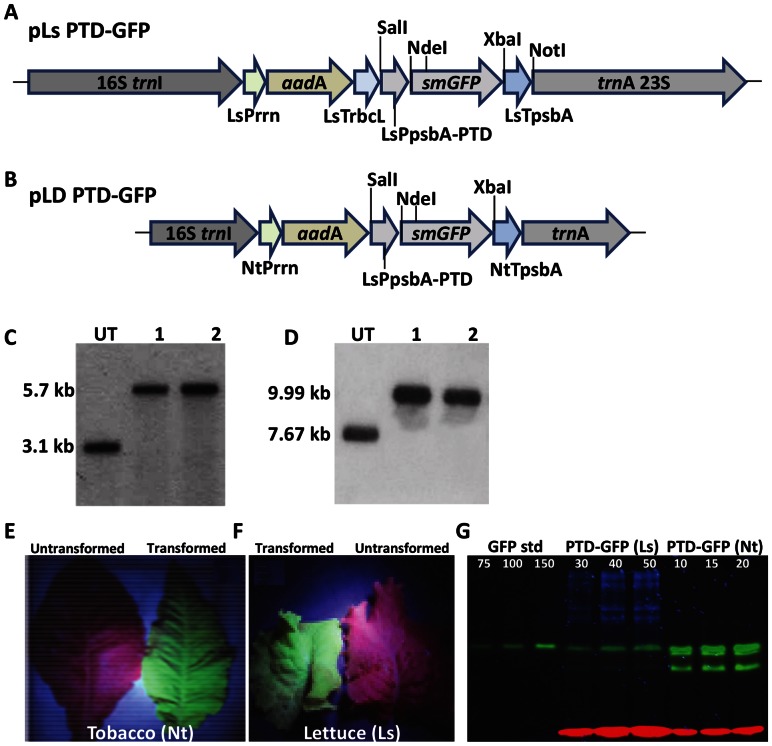
Regeneration of transplastomic PTD-GFP tobacco and lettuce plants. Schematic representation of lettuce (A), tobacco (B) chloroplast transformation vectors. LsPrrn, *Lactuca sativa* rRNA operon promoter and GGAG ribosome binding site; aadA, aminoglycoside 3′-adenylyltransferase gene; smGFP, soluble modified green fluorescent protein; LsTrbcL, 3′ untranslated region (UTR) of *Lactuca sativa* rbcL gene; LsPpsbA-PTD, promoter and 5′ UTR of *Lactuca sativa* psbA gene fused to protein transduction domain (PTD, amino acid sequence – RHIKIWFQNRRMKWKK) of pancreatic and duodenal homeobox factor-1 (PDX-1); LsTpsbA, 3′ UTR of *Lactuca sativa* psbA gene; NtPrrn, *Nicotiana tabacum* rRNA operon promoter and GGAG ribosome binding site; NtTpsbA, 3′ UTR of *Nicotiana tabacum* psbA gene; 16S trnI in pLs PTD-GFP, the homologous long flanking sequence from *Lactuca sativa* chloroplast genome containing 16S 3′ end sequences and full length trnI gene; trnA 23S in pLs PTD-GFP, the homologous long flanking sequence from *Lactuca sativa* chloroplast genome containing full length trnA gene and 5′ end of the 23S ribosomal RNA subunit; 16S trnI in pLD PTD-GFP, the homologous flanking sequence from *Nicotiana tabacum* chloroplast genome containing 16S 3′ end sequences and full length trnI gene; trnA in pLD PTD-GFP, the homologous flanking sequence from *Nicotiana tabacum* chloroplast genome containing full length trnA gene. (C and D) Southern blots of PTD-GFP lettuce and tobacco plants. UT, Untransformed; 1 and 2, transplastomic lines. (E and F) GFP fluorescence in transplastomic PTD-GFP tobacco and lettuce leaves observed under blue light or chlorophyll fluorescence in untransformed control. (G) Non-denaturing gel for quantification of PTD-GFP. GFP standard protein was loaded at indicated concentration (ng). Total soluble proteins from transplastomic plants were extracted three times from independent lines, loaded as indicated (μg) and quantified using densitometry.

Two new chloroplast transformation vectors were designed for expressing PTD-GFP in lettuce or tobacco (Figure 1A and 1B) and GFP-RC101 vector from a previous study was used [15]. The design of chloroplast transformation vectors used here is similar to previous studies in our lab [15], [20]. PTD is the protein transduction domain of PDX1 (pancreatic and duodenal homeobox factor-1) [19] and Retrocyclin-101 (RC101) is an antimicrobial peptide. Both were fused in frame with GFP for expression in chloroplasts, regulated by the *psb*A promoter and its 5′ and 3′ untranslated regions to attain higher levels of expression.

Transplastomic tobacco and lettuce expressing PTD-GFP plants were regenerated as described previously [20], [22]. PCR analysis showed that the transgene integration occurred at specific site of chloroplast genome (data not shown). Southern blot analysis confirmed homoplasmy and site-specific integration into the chloroplast genome. Total plant DNA digestion with *Sma*I and *Hind*III for the lettuce and tobacco respectively, generated 3.1 kb or 7.67 kb in untransformed and 5.7 kb or 9.99 kb in transplastomic lines after hybridization with the *trn*I-*trn*A flanking sequence [^32^P]-labeled probe (Figure 1C and 1D). Furthermore, the absence of 3.1kb or 7.67 kb in the transplastomic lines established that homoplasmy was attained. In comparison to untransformed plants, the phenotype of transplastomic lines appeared to be normal with typical flowering and seed setting. Observation of transplastomic PTD-GFP tobacco and lettuce plants under UV light showed high GFP fluorescence while only chlorophyll fluorescence was observed in untransformed plants (Figure 1E and 1F). The expression of PTD-GFP protein in tobacco and lettuce was further confirmed by the green fluorescence in protein extracts separated by native polyacrylamide gel electrophoresis and observed under UV light. The visualization of intense green fluorescence indicates that PTD-GFP fusion protein accumulated at high levels (Figure 1G). The expression levels of PTD-GFP transplastomic plants were estimated to be approximately 2% (±0.1) and 9.7% (±0.9) TSP (total soluble protein) for lettuce and tobacco respectively based on densitometric data (at different developmental stages).

### Release of GFP protein from chloroplasts under biotic stress

**Figure 2:**
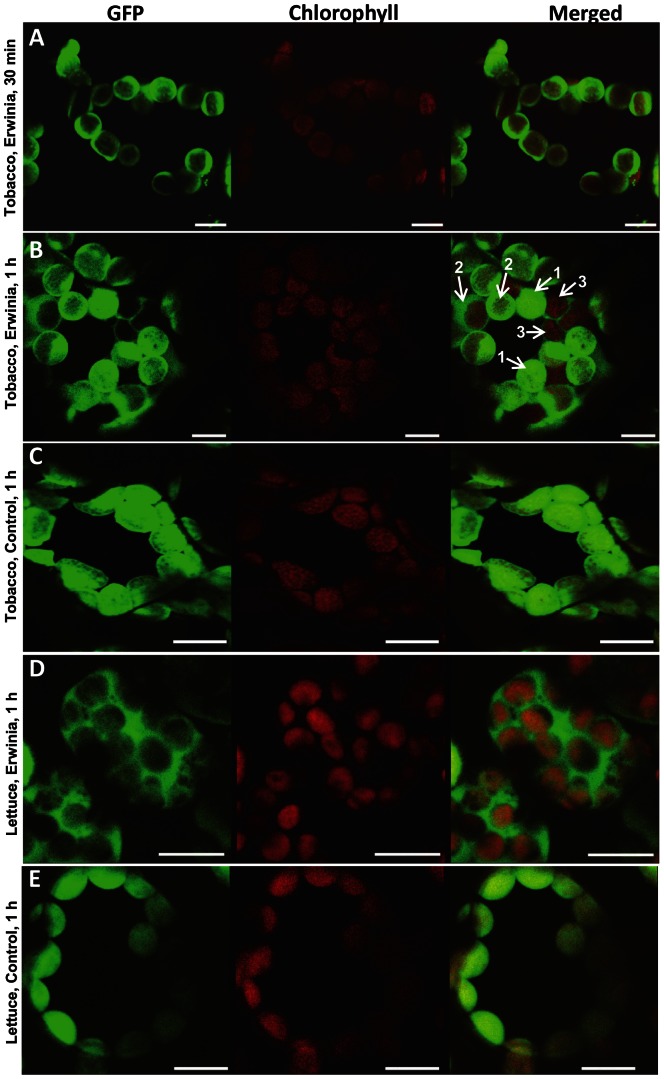
Evaluation of PTD-GFP fluorescence by confocal microscope in tobacco and lettuce leaf discs after *E. carotovora* infection. Leaf discs (9 mm in diameter) made using cork borer were infected with *E. carotovora* (OD_600_ = 0.2) in a multi-well culture plate and plant cells were imaged by confocal microscopy. Representative images are from 67 stored images out of 189 observations of cells near the periphery of tobacco leaf discs incubated with *E. carotovora* for 30 min (A) or 1 hr (B). Different stages of GFP release were indicated by arrows and numbers. Arrow #1 represents very early stage of GFP release, showing negligible loss of GFP fluorescence. Arrow #2 represents intermediate step of GFP release demonstrating both GFP and chlorophyll fluorescence in chloroplasts. Arrow #3 represents late stage of GFP release outside of chloroplasts with detection of only red chlorophyll fluorescence. (C) Representative image from 70 stored images out of 194 observations of leaf discs of tobacco without *E. carotovora* inoculation. (D) Representative images are from 74 stored images out of 191 observations of cells near the periphery of lettuce leaf discs incubated with *E. carotovora* for 1 hr. (E) Representative images are from 68 stored images out of 153 observations of lettuce leaf discs without *E. carotovora* inoculation. Each experiment was repeated five times and each time two leaves from at least two different plants were used to make 6 discs per treatment. Bars represent 10 µm.

**Figure 3:**
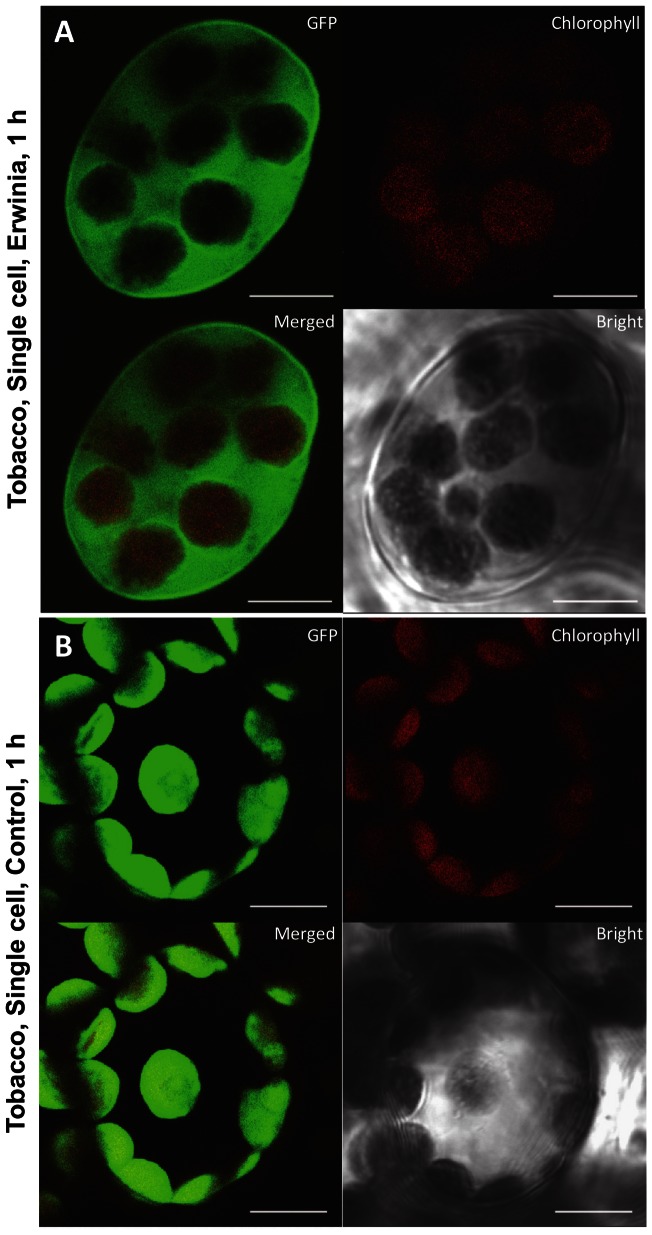
Evaluation of PTD-GFP fluorescence by confocal microscope in individual cells of tobacco leaf discs with or without *E. carotovora* infection. (A) An enlarged view of a single tobacco cell showing intact chloroplasts fully releasing GFP after 1 hr of *E. carotovora* incubation. (B) An enlarged view of a single cell from tobacco leaf discs without *E. carotovora* incubation. Images of GFP fluorescence, chlorophyll fluorescence, merged and bright field images are provided.

Tobacco and lettuce transplastomic leaf discs were inoculated with *Erwinia carotovora* and observed under the confocal microscope. For Erwinia infection, infiltration of the leaf with the pathogen was not efficient to achieve the infection (data not shown). Therefore, leaf discs were used to enhance Erwinia infection efficiency as reported previously [30]. In tobacco transplastomic lines, GFP was found to be localized towards the chloroplast envelope and further released into the cytoplasm from intact chloroplasts within 1 hr of infection with *E. carotovora* while red chlorophyll fluorescence was detected in chloroplasts. Also, an intact central vacuole was observed during the release of GFP (Figure 2A and 2B). Soon after Erwinia infection (within 30 minutes), most of the chloroplasts showed GFP move from the center of chloroplasts towards the envelope (Figure 2A). Later on, in a few chloroplasts GFP was fully released with detection of only red chlorophyll fluorescence (Figure 2B, arrow #3). At the same time, some chloroplasts within the same cell showed negligible loss of GFP fluorescence (Fig 2B, arrow #1). Intermediate steps of GFP release between these two stages with both GFP and chlorophyll fluorescence are shown with arrow #2 in Fig 2B. In more advanced stages of Erwinia infection, all chloroplasts showed only chlorophyll fluorescence and the GFP was observed only outside chloroplasts, after one hour in leaf discs (Figs 2D, 3A). Representative images showing GFP fluorescence signal are from 67 stored images out of 189 observations of cells near the periphery of tobacco leaf discs incubated with *E. carotovora* for 30 min or 1 hr (Figures 2A and 2B). In case of transplastomic plants without *E. carotovora* infection, GFP was detected only within intact chloroplasts (Figure 2C), indicating that GFP release is not due to the cutting of the disc or any other mechanical damage. The enlarged view of single cell of tobacco leaf discs without *E. carotovora* inoculation is also provided for comparison (Figure 3B). The same pattern of GFP movement was also observed in lettuce transplastomic leaf discs upon infection with *E. carotovora* (Figure 2D), while GFP in uninfected transplastomic lines was inside the intact chloroplasts (Figure 2E). Reproducibility of these observations is reassured by examination of GFP fluorescence signal from 60–75 stored images out of 150–200 observations of cells near the periphery of lettuce leaf discs incubated with *E. carotovora* for 1 hr or without infection (Figure 2D and 2E). Each experiment was repeated five times and each time two leaves from at least two independent lines were used to prepare discs for treatment. Though only representative confocal images are presented in figures, all different stages of GFP movement could be observed at each time point or within each cell. But the representative images were selected from the majority of images showing similar phenomenon at that time point. To ensure the reliability of phenomenon observed under the confocal microscope, all observations were recorded and images were stored.

**Figure 4:**
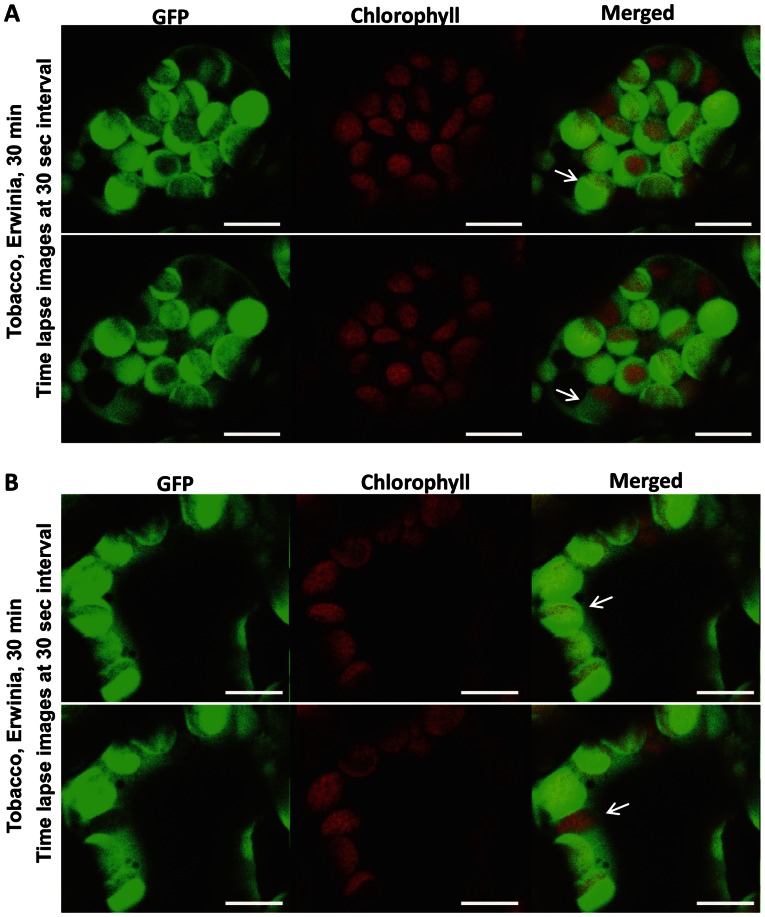
Release of GFP from intact chloroplasts viewed by time lapse confocal microscopic imaging of PTD-GFP tobacco leaf discs after *Erwinia carotovora* infection. Leaf discs of ∼9 mm diameter were inoculated with *E. carotovora* (OD_600_ = 0.2) culture in a multi-well plate for 30 min and plant cells were observed by time lapse imaging at 30 second intervals under confocal microscope. (A & B) Arrow in Figure A top panel points to a chloroplast with most of GFP within that chloroplast but the bottom panel (image taken at 30 sec interval) shows more than 50% of GFP released outside the chloroplast. Likewise, in figure 4B, top panel, arrow shows GFP within chloroplasts but after 30 sec interval, all of the GFP has been released with only red chlorophyll fluorescence in the bottom panel. Bars represent 10 µm.

In order to evaluate intactness of chloroplasts and to make sure GFP observed in the cytoplasm outside of chloroplasts after Erwinia infection is not due to the lysis of chloroplasts, we observed chloroplasts under bright field and performed time lapse confocal microscopy. The outline of intact chloroplasts is quite evident after complete release of all GFP (Figure 3A). The complete movement of GFP from intact chloroplast to cytosol under stress conditions was repeatedly observed after one hour. The initial time lapse point showed GFP inside the intact chloroplasts within the cell (Figure 4A and 4B; upper panel). During the subsequent time lapse points, GFP was gradually released from the intact chloroplasts and GFP fluorescence was either undetectable, decreased or a proportion of the chloroplasts are releasing GFP (Figure 4A and 4B; lower panel). At the same time, GFP fluorescence was detected in cytoplasm outside of intact chloroplasts. Simultaneously, chloroplasts with intense GFP fluorescence or with only chlorophyll autofluorescence representing chloroplasts with no or complete release of GFP were also observed. Arrows in figure 4 show specific chloroplasts within a cell that are in the process of releasing GFP. The decrease of GFP fluorescence in the chloroplast is not associated with GFP degradation caused by possible acidic pH because the pH of stroma increases from 7 in dark to 8 in light [31]. In this study, all treatments (biotic and abiotic stress) were done under the light. Chla red fluorescence further showed that chloroplasts remained intact during the release of GFP. The Chla fluorescence has been used as a simple and non-invasive tool to monitor chloroplast functions in vivo and in vitro [32]. Abundant Chla fluorescence after the release of GFP (Figure 2B, 2D, 4A and 4B) shows that thylakoid membranes are intact and fully functional to carry out photochemical electron transport and photosynthesis.

### Role of Reactive Oxygen Species (ROS) under biotic/abiotic stress

**Figure 5:**
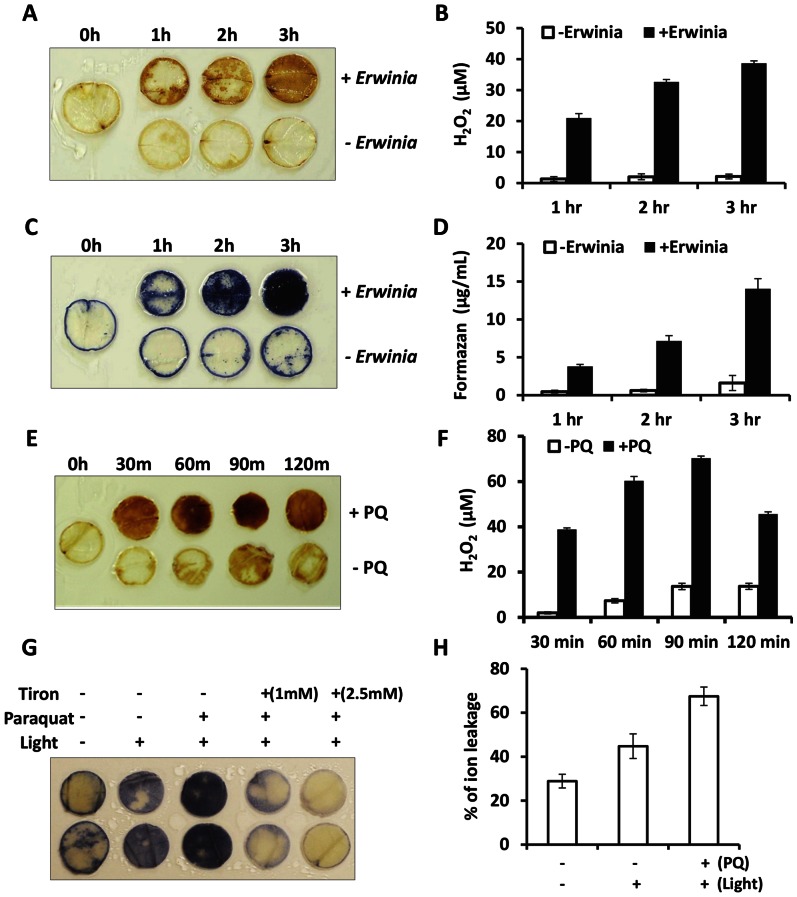
Evaluation of ROS in tobacco after biotic (Erwinia) and abiotic (paraquat) stress by DAB, NBT and ion leakage. Leaf discs were incubated in water for 2 hr under dim light and were subjected to biotic, *E. carotovora* (OD_600_ = 0.2), or abiotic stresses. Twenty leaf discs were used for each treatment except for ion leakage studies, six leaf discs were evaluated. (A, C) DAB and NBT staining after *E. carotovora* infection. (B, D) Quantification of generated H_2_O_2_ and O2·^−^. Formazan, precipitates formed from reduction of NBT by superoxide anions. (E) DAB staining after paraquat treatment. (F) Quantification of generated H_2_O_2_ after paraquat treatment. (G) NBT staining of pretreated leaf discs with or without Tiron (scavenger of superoxide anion), after paraquat treatment. (H) Effect of paraquat on ion leakage. Each experiment was repeated three times from independent lines and at least five different plants were used to prepare leaf discs. The error bars are represented as mean ± SD.

To investigate the role of Reactive Oxygen Species (ROS) in protein export out of chloroplasts after *E. carotovora* infection, hydrogen peroxide and superoxide anion were evaluated with DAB (3, 3′-diaminobenzidine tetrahydrochloride hydrate) and NBT (nitroblue tetrazolium), respectively. Oxidation of DAB and reduction of NBT were detected within 1 hr after infection and gradually increased until 3 hr. In contrast, there were no significant color changes in leaf discs that were not treated with *E. carotovora* (Figure 5A and 5C). To determine the generated ROS quantitatively, precipitated DAB and NBT were extracted and quantified based on the standard curves. The production of H_2_O_2_ and O_2_•^−^ increased with the duration of bacterial infection, up to 18 fold and 7 fold when compared to control, respectively (Figure 5B and 5D). These data indicate that the generation of ROS was triggered in tobacco leaf discs by the infection of *E. carotovora.*

To investigate the role of ROS in protein release out of chloroplasts under abiotic stress, paraquat (PQ), superoxide radical inducer [33], was tested. The production of H_2_O_2_ reached its maximum of ∼60 μM at 90 min after treatment but maximum fold increase, almost 20-fold, occurred at 30 min after treatment (Figure 5E and 5F). To investigate the correlation between protein release from chloroplasts under abiotic stress and superoxide involvement, Tiron, scavenger of superoxide anion, was also tested. Superoxide was distinctly detectable even with no PQ treatment under high light conditions. Upon paraquat treatment, the color was deepened due to increase of superoxide radical by paraquat. Formazan precipitation from reduction of NBT by O_2_•^−^, however, was dramatically reduced upon Tiron treatment (Figure 5G). Taken together, these results showed that ROS (O_2_•^−^ and H_2_O_2_) was generated by both biotic and abiotic stress and superoxide production could be inhibited by the treatment of Tiron. In order to assure reproducibility, at least twenty leaf discs were used for each treatment and repeated three times independently. At least five independent lines were used to prepare leaf discs.

**Figure 6:**
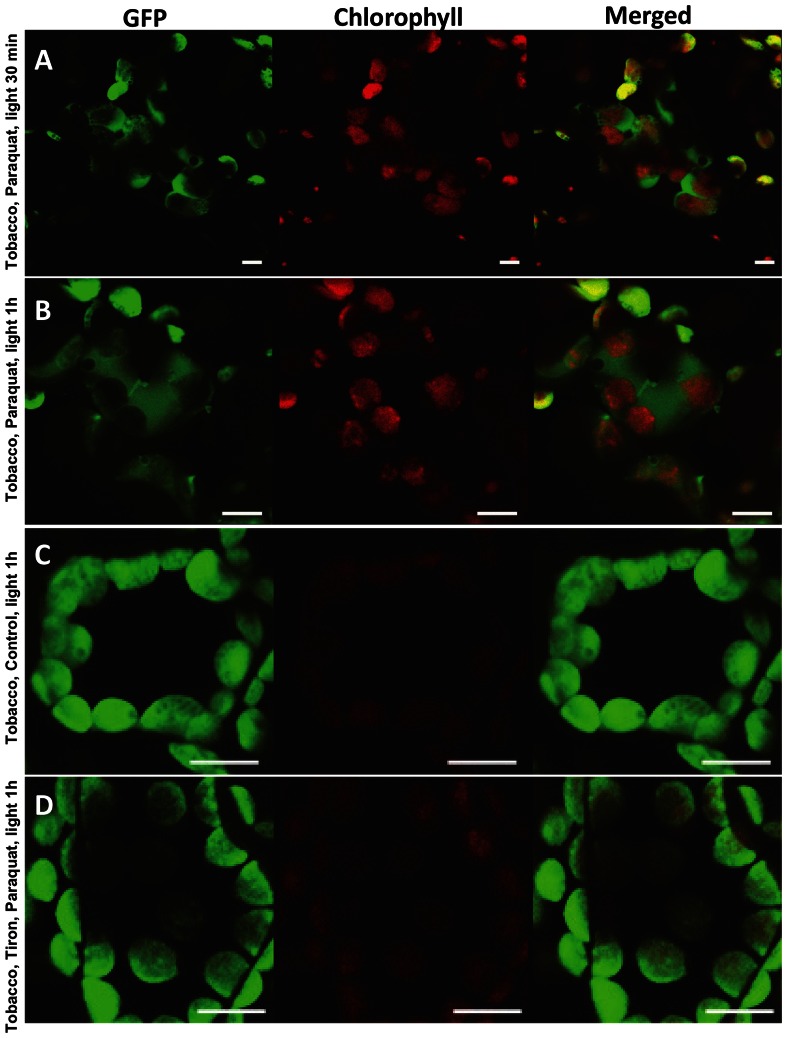
Visualization of PTD-GFP fluorescence in tobacco after paraquat treatment. Leaf discs were vacuum infiltrated in 0.1% Tween 20 with or without paraquat (16 µm) for 2 min, transferred to water after rinsing. Representative images are from 66 stored images out of 172 observations of cells illuminated for (A) 30 min or (B)1 hr. (C) Control is among 43 stored images out of 157 observations, without paraquat treatment. (D) Representative image is from 37 stored images out of 143 observations of Tiron (2.5 mM) pretreated leaf samples, followed by paraquat treatment and illumination for 1 hr. Each experiment was repeated five times and each time two leaves from at least two independent lines were used to make 6 discs per treatment. Bars represent 10 µm.

PQ accepts electrons from photosystem I, and the resulting free radical form reacts with oxygen to produce superoxide [34]. In order to investigate the PQ induced oxidative stress on the integrity of chloroplast envelope membranes, ion leakage was measured after treatment with PQ in the light. The ion leakage of the PQ treated samples increased by 51% in the light and 134% in the presence of PQ and light when compared with respective controls in the dark (Figure 5H). This means that membrane integrity was disturbed by light and more severely by PQ. Ion leakage in the PQ treated samples in the light suggests changes in the chloroplast envelope membrane integrity and facilitates the release of proteins from chloroplasts. For the ion leakage studies, six leaf discs were evaluated for each treatment. In order to investigate this further, leaf discs were treated with PQ and observed under the confocal microscope. In this case, export of GFP was more rapid than infection with *E. carotovora*. Within 30 min of PQ treatment, GFP was localized towards the envelope and outside chloroplasts (Figure 6A and 6B) while in control GFP was localized within intact chloroplasts (Figure 6C). In order to assure reproducibility, GFP fluorescence signal was examined in >170 images (66 stored images) of cells illuminated for 30 min or 1 hr (Figure 6A and 6B). We examined >150 images (43 stored images) of cells without PQ treatment (Figure 6C). This observation suggests the involvement of ROS in protein export. Tiron has been used to inhibit generation of superoxide radical. In order to study the inhibition of O_2_•^−^ production by Tiron, pretreated leaf with Tiron was subjected to PQ treatment. Both confocal microscope and NBT studies revealed that ROS generated by abiotic stress induced the release of GFP from chloroplasts and Tiron successfully blocked the release of GFP from chloroplast. The representative image is from 37 stored images out of 143 observations (Figure 6D). Each experiment was repeated five times and each time two leaves from at least two independent lines were used to make 6 discs per treatment.

### GFP-Retrocyclin101 showed resistance to *E. carotovora* infection

**Figure 7:**
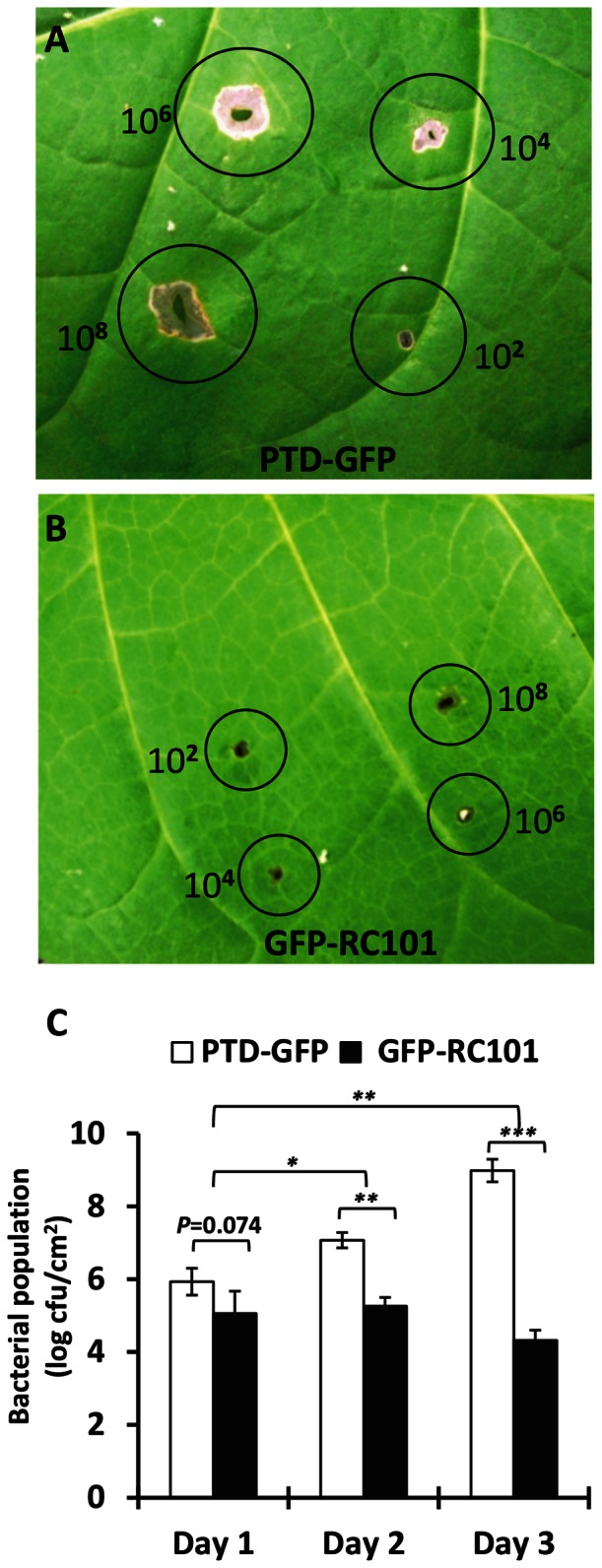
In planta bioassays of PTD-GFP and GFP-RC101 tobacco. Tobacco leaves were injected with *E. carotovora* (10^8^, 10^6^, 10^4^ and 10^2^ cells) using a syringe with a precision glide needle. Photos were taken 5 dpi (day post inoculation). Tobacco leaves of PTD-GFP (A) or GFP-RC101 (B) infected with *E. carotovora*. (C) Quantitation of bacterial colonization after *E. carotovora* infection. *P<0.05, **P<0.001, and ***P<0.0001. Each experiment was repeated three times using independent lines and all values represent means of three replicates with standard deviations shown as bars.

To investigate the mechanism of antimicrobial peptides expressed within chloroplasts, GFP-RC101 and PTD-GFP leaves were challenged with *E. carotovora* by syringe injection method [15]. The symptoms of damage were observed on leaves of PTD-GFP plants near the site of inoculation one day after infection with *E. carotovora*. On the third day after infection, leaves of PTD-GFP showed necrosis adjacent to the inoculation point even with very low density (10^2^ bacterial cells) of *E. carotovora* infection (Figure 7A), whereas GFP-RC101 tobacco leaves showed negligible necrosis even with inoculation of 10^8^ bacterial cells (Figure 7B). The bacterial population in the inoculated area of plant leaves was measured as described previously [15]. The bacterial populations in GFP-RC101 and PTD-GFP tobacco leaves were around 1×10^5^ cfu/cm^2^ one day post inoculation (dpi). However, the total population in the PTD-GFP leaf soared up to 9×10^8^ cfu/cm^2^ when the PTD-GFP was inoculated with *E. carotovora* on 3 dpi (Figure 7C). *E. carotovora* populations were less than 1×10^5^ cfu/cm^2^ in the GFP-RC101 tobacco leaves on 3 dpi (Figure 7C). Also, no obvious necrosis symptoms were observed on any of the GFP-RC101 plants. These data show that the GFP-RC101 plants are unaffected by *E. carotovora* infection. Each experiment was repeated three times using independent lines and all values represent means of three replicates with standard deviations shown as bars.

### Release of GFP from PTD-GFP and RC101-GFP plants after in planta infection with *E. carotovora*

**Figure 8:**
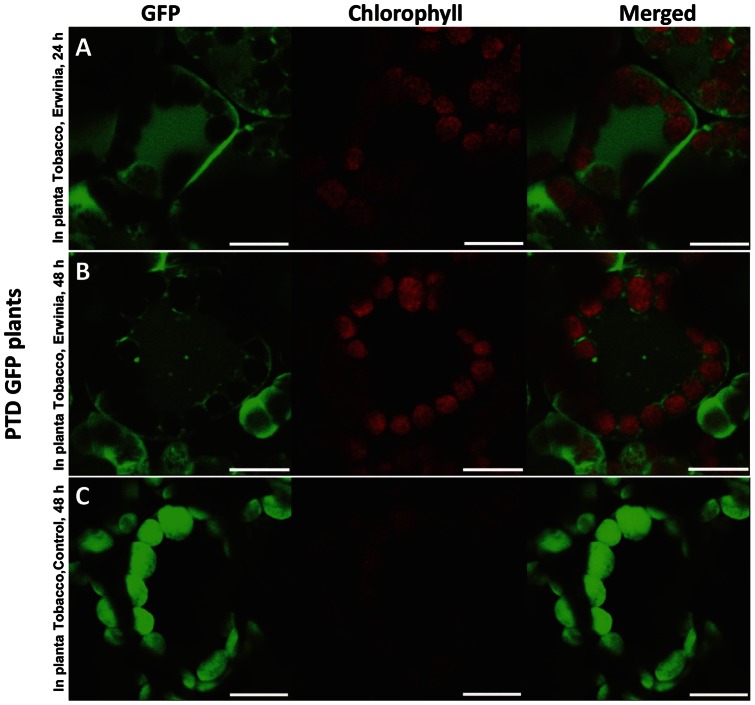
In planta PTD-GFP transplastomic tobacco leaves after *E. carotovora* infection, imaged by confocal microscope. Bacterial suspension (1.0×10^5^ cells) of *E. carotovora* was injected into the tobacco leaves with a syringe. The infected area (1 cm^2^ disk) of individual plant was punched-out from the leaves (five leaf discs for each point) and analyzed by confocal microscopy after 24 or 48 hr of *E. carotovora* infection. Representative images are from 57 stored images out of 175 observations of PTD-GFP chloroplasts after 24 (A) or 48 hr (B) of E. carotovora infection. (C) Representative images are from 50 stored images among 167 observations of PTD-GFP leaf cells without *E. carotovora* infection. Bars represent 10 µm.

**Figure 9:**
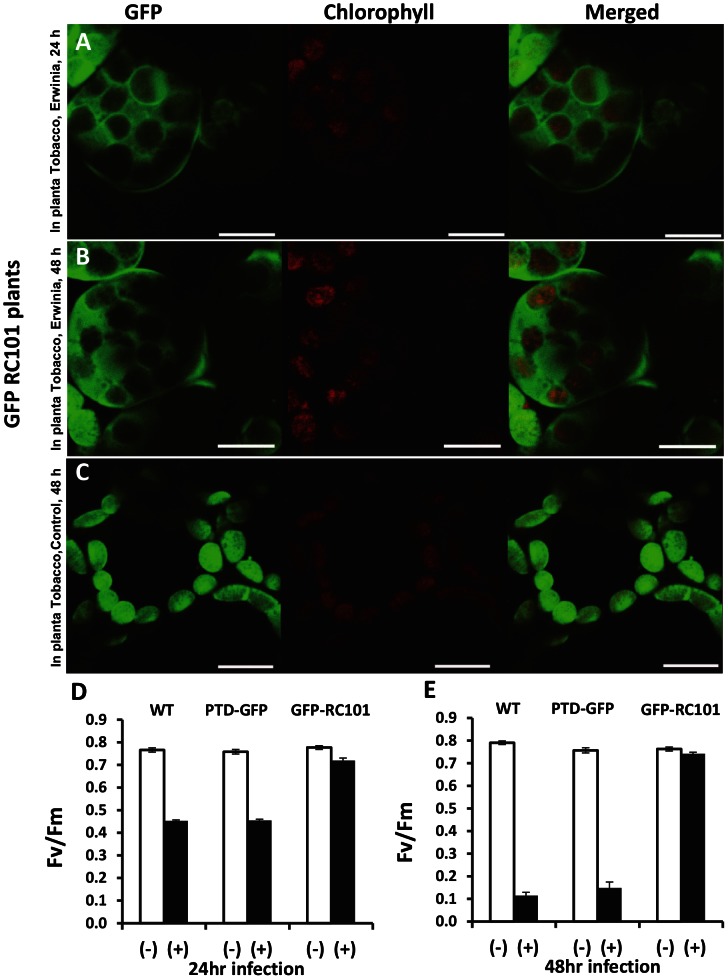
In planta GFP-RC101 transplastomic tobacco leaves after *E. carotovora* infection, imaged by confocal microscope and their photosynthetic efficiency measured by PAM fluorometer. Bacterial suspension (1.0 ×10^5^ cells) of *E. carotovora* was injected into the tobacco leaves with a syringe. The infected area (1 cm^2^ disk) of individual plant was punched-out from the leaves (five leaf discs for each point) and analyzed by confocal microscopy after 24 or 48 hr of *E. carotovora* infection. Representative images are from 62 stored images out of 189 observations of GFP-RC101 after 24 (A) and 48 hr (B) of *E. carotovora* infection. (C) Representative images are from 41 stored image out of 156 observations, GFP-RC101 leaf cells without *E. carotovora* infection. Each experiment was repeated at least three times using independent lines. Bars represent 10 µm. Photosystem II maximum efficiency of untransformed (WT), PTD-GFP and GFP-RC101 plants without (white bar) and with Erwinia infection (black bar) after 24 hr (D) and 48 hr (E).

The response of plant cells in PTD-GFP and GFP-RC101 whole plant leaves to the *E. carotovora* was observed by confocal microscope. In this experiment, leaf pieces were made after 24 and 48 hr infection of leaves in planta by Erwinia and mounted on glass slides to observe GFP fluorescence. After 24 hr of infection by *E. carotovora*, the GFP movement out of chloroplasts could be observed both in the PTD-GFP and the GFP-RC101 samples (Figure 8A and 9A). However, the chloroplasts in the control were intact and no GFP movement out of chloroplasts was observed (Figure 8C and 9C). The GFP fluorescence was also detected as small spherical bodies in the cells (Figure 8B). The GFP movement out of chloroplast in the PTD-GFP was much higher than the GFP-RC101 leaves after 48 hr of *E. carotovora* infection (Figure 8B and 9B). The most significant difference was that the area of GFP movement out of chloroplast in the PTD-GFP was twice as large as the area in the GFP-RC101 from the point of inoculation (center of *E. carotovora* infection area, data not shown). The representative images showing GFP signal are from 57 stored images out of 175 observations of the PTD-GFP chloroplasts after 24 or 48 hr of *E. carotovora* infection (Figure 8A and 8B). The representative control images are from 50 stored images among 167 observations of the PTD-GFP leaf cells without *E. carotovora* infection (Figure 8C). The representative images showing GFP signal are from 62 stored images out of 189 observations of GFP-RC101 after 24 and 48 hr of *E. carotovora* infection (Figure 9A and 9B). The representative control images are from 41 stored images out of 156 observations of the GFP-RC101 leaf cells without *E. carotovora* infection (Figure 9C). Each experiment was repeated at least three times.

Following the release of GFP from intact chloroplasts after Erwinia infection, chloroplasts still showed intense red chlorophyll fluorescence (Figure 8A, 8B, 9A and 9B) indicating intact thylakoid membranes with ability to perform photochemical electron transport and photosynthesis. To evaluate the intactness of photochemical electron transport chain within the chloroplasts, the maximum quantum yield of photosystem II was measured using portable chlorophyll fluorometer (PAM-2100). The Fv/Fm values were taken in plant leaves of untransformed, PTD-GFP and GFP-RC101 transplastomic tobacco plants after 24 and 48 hr of Erwinia infection (Figure 9D and 9E). The corresponding uninfected leaves were used as respective controls. Typically, Fv/Fm value for non-stressed leaves from plants growing in field is around 0.8 [35] whereas in our measurements Fv/Fm values ranged from 0.74–0.8 in non-infected leaves. No significant difference in Fv/Fm values of the GFP-RC101 leaves was observed between 24 hr and 48 hr (ranging from 0.70 to 0.75) after Erwinia infection (Figure 9D and 9E) due to the release of antimicrobial peptide (GFP-RC101) from intact chloroplasts. In contrast, the infection by Erwinia lowered Fv/Fm values by up to 41% in the untransformed and the PTD-GFP plants when compared with their respective uninfected controls after 24 hr of treatment (Figure 9D). However, a decrease of only 7.4% was observed in the GFP-RC101. The Fv/Fm values dropped ∼85% after 48 hr of Erwinia infection in the untransformed and the PTD-GFP plants whereas the drop was only 2.7% in the GFP-RC101 (Figure 9E). These results suggest that the GFP fusion protein was released from intact fully functional chloroplasts during the early response to Erwinia infection.

## Discussion

Several environmental factors influence metabolic functions and plastids must direct nuclear gene expression and protein flow via retrograde signaling. The up or down regulation of nuclear-encoded photosynthetic genes takes place due to the changes in chloroplast redox status [36]. Apart from its vital role, retrograde signaling also significantly controls a plant's adaptive response to different stresses [37]. Despite extensive research on retrograde signaling, the current understanding remains limited and the suggested cytosolic signaling pathways and the presumed organellar signaling molecules remain obscure [38]. Reactive oxygen species holds substantial attention as retrograde signal molecules, mainly because of their active control [39].

**Figure 10:**
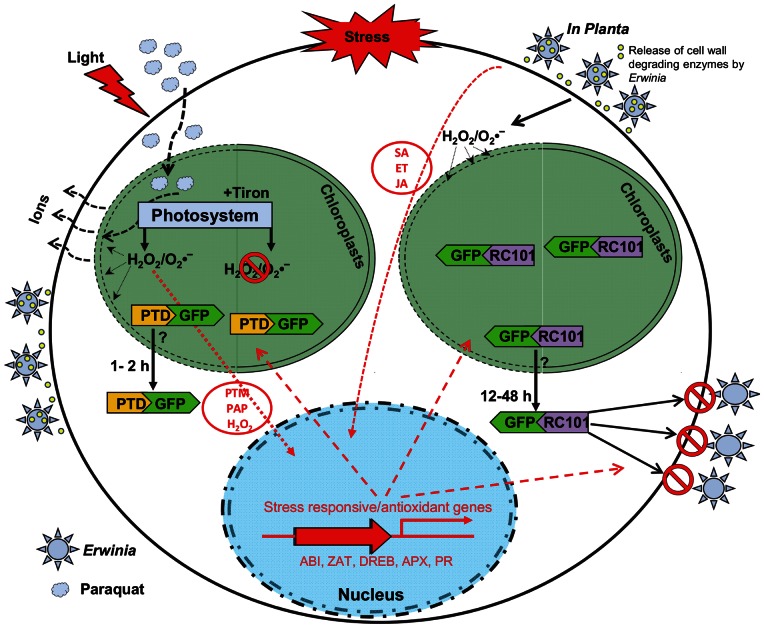
A model showing GFP export from chloroplasts of transplastomic plants under stress. *Erwinia carotovora* and paraquat generate superoxide anions and H_2_O_2_. These reactive oxygen species (ROS) oxidize chloroplast lipid membranes and alter chloroplast envelope, increasing ion leakage. Consequently stromal proteins are exported from the chloroplasts into the cytoplasm. The signal molecules include H_2_O_2_, PTM (a chloroplast envelope-bound plant homeodomain transcription factor under photo-oxidative stress), PAP (3′-phosphoadenosine 5′-phosphate under high light or drought stress) are present in the chloroplast under stress conditions and communicate with nucleus to regulate stress responsive and antioxidant genes. ABI, abscisic acid insensitive transcription factors; DREB, dehydration responsive element binding proteins; ZAT, zinc finger transcription factors; APX, ascorbate peroxidase genes; PR, pathogenesis related genes; SA, salicylic acid; JA, jasmonic acid; and ET, ethylene. Early events caused by biotic/abiotc stress treatments in this study are represented in black solid and dotted arrows. Downstream signaling events following the outburst of ROS, which have been reported in previous studies by other groups, are indicated in red solid and dotted arrows, and letters. Yellow dots represent cell wall degrading enzyme secreted by Erwinia.

*E. carotovora* infects a large number of plants via secretion of cell wall degrading enzymes resulting in induction of signaling pathways, oxidative burst and host defense mechanism [40], [41]. Paraquat treatment also generates ROS including superoxide anions and H_2_O_2_. These ROS oxidize chloroplast lipid membrane leading to changes in the chloroplast membrane integrity and ion leakage (Figure 10). As a result stromal proteins move out from the chloroplasts to the cytoplasm. These proteins, including transcription factors, trigger nuclear stress response and activate antioxidant genes to protect the host plant [9]. Similarly, paraquat treatment in the light increased ion leakage.

Downstream events describing nuclear gene expression in response to biotic or abiotic stress triggered by ROS signaling is not the focus of this study but has been extensively documented by previous studies. For example, changes in nuclear gene expressions upon paraquat treatment resulted in increase in production of antioxidants, cellular protection and detoxification [42], [43]. ROS accumulated in chloroplasts generated by paraquat treatment induced signaling pathways common to several abiotic stress responses [43]. Similarly, ROS production is part of the initial multifaceted responses generated after pathogen attack. ROS interaction with other plant signaling molecules such as salicylic acid (SA), jasmonic acid (JA) and ethylene (ET) activate defense signals and responses [44]. Consequently, there is an increase in magnitude of functionally different proteins and metabolites. A set of plant cell wall degrading enzymes consisting of proteases, cellulases and pectinases are the main virulence factors which trigger SA independent and JA/ET dependent defense signaling [41], [45]. Some strains of *Erwinia carotovora* generate heat stable virulence factors known as harpins, which collectively induce SA-dependent and JA/ET-dependent signaling pathways [45]. The infiltration of plants with the purified harpins resulted in enhanced ion leakage and along with other virulence factors generated higher levels of ROS [45]. Also, harpins from *Pseudomonas syringae* have been implicated in intensification of ROS production and activation of various signaling pathways [45], [46]. Therefore, this study focused only on early events of biotic/abiotic stress which are poorly understood and did not focus on well-known downstream signaling events.

In this study, GFP was seen in the cytoplasm after Erwinia infection and paraquat treatment. Tiron, an inhibitor of superoxide anions, minimized the paraquat effect. Recent studies showed that retrograde signal molecules (PTM, a chloroplast envelope-bound plant homeodomain transcription factor under photo-oxidative stress; PAP, 3′-phosphoadenosine 5′-phosphate under high light or drought stress, and H_2_O_2_ under high light) are present in the chloroplast under stress conditions and communicate with the nucleus (Figure 10) [9], [47], [48]. As a result of the release of signal molecules from chloroplast, various transcription factors are upregulated such as abscisic acid insensitive (ABI) transcription factors, dehydration responsive element binding proteins (DREB) and zinc finger transcription factors (ZAT) in the nucleus [48]–[50]. Furthermore, many stress and defense-related genes such as ascorbate peroxidase (APX) and pathogenesis related (PR) proteins are activated as explained in Figure 10 [47], [51].

Several mechanistic insights could be provided for the release of proteins from intact chloroplasts under biotic or abiotic stress based on the published literature. Data provided in this manuscript on time lapse images, maximum quantum yield of photosystem II and Chla fluorescence confirms that chloroplasts remain intact during release of GFP. Separation of thylakoid membranes during aging or senescence dramatically reduces Chla fluorescence [52]. Chla fluorescence at room temperature emanating from chloroplasts reflects photoreduction of electron transport carriers and intactness/integrity of chloroplasts and thylakoid membranes [53], [54]. Furthermore, the time lapse images showed the intactness of chloroplasts and confirmed that GFP observed in the cytoplasm around the chloroplasts did not originate by the lysis of chloroplasts but instead is released from the intact chloroplasts during biotic stress.

Several previous reports provide indirect evidence for the release of proteins or large molecules from chloroplasts. For example, when Tic40, a protein within the import complex localized in the inner plastid envelope was expressed within chloroplasts via the chloroplast genome, all other inner membrane proteins encoded by the nuclear genome were highly upregulated [13], suggesting retrograde signal transduction is initiated by Tic40 in healthy plants under no stress. Antimicrobial peptides expressed through the chloroplast genome conferred protection against bacterial or viral pathogens [14], [15] by their release from intact chloroplasts. Furthermore, expression of biomass hydrolysis enzymes within chloroplasts, again conferred high levels of protection to plant pathogens [16], indicating a hyper-sensitive response triggered by proteins expressed within plastids. A chloroplast localized receptor interacting protein (NRIP1) was demonstrated to interact with the P50 helicase in the cytoplasm during tobacco mosaic virus (TMV) infection [17]. The majority of *Pseudomonas syringae* secreted proteins have chloroplast targeting signal sequences, requiring retrograde signaling to the nucleus in order to trigger defense response [18]. In this study, we show that release of the antimicrobial peptide expressed within chloroplasts protects the transplastomic plants from Erwinia infection. All these observations suggest that large molecules (peptides/proteins) could leave chloroplasts and play a significant role in retrograde signaling.

ROS generated in chloroplasts function as retrograde signals by communicating with nucleus to upregulate production of antioxidant enzymes and by amending the photosynthetic machinery for effective light harvesting [55], [56]. The ROS are generated from not only abiotic stress, but also biotic stress. Polyunsaturated fatty acids produce multiple peroxide molecules by chain reactions caused by ROS [33]. These small molecules generated by ROS induced by Erwinia infection can freely penetrate the envelope or be transported by membrane transporter to send signals to the nucleus. But when more ROS is accumulated by sustained stress, more lipid damage occurs and proteins inside chloroplasts are released to send signals to the nucleus like NRIP [17] in addition to peptide and lipid derivatives. Therefore, ROS production has dual roles in signal transduction and increase of membrane leakiness. In our study we provide direct evidence for this process using GFP transplastomic plants that production of superoxide and hydrogen peroxide under biotic and abiotic stress releases GFP from chloroplasts, in a timely manner (Figure 2B, 2D and 6B). At an early stage of Erwinia infection (<30 min), detected location of GFP signal was different between chloroplasts within the same cell, suggesting that the concentration of ROS could be different among chloroplasts within the same cell (Figure 2B). However, complete release of the GFP signal was observed in most of chloroplasts after an hour (Figure 2D and 3A) due to continued accumulation of ROS caused by Erwinia infection, as shown in this study (Figure 5) and described in a previous report [57]. However, GFP release could be minimized or eliminated by blocking ROS. Moreover, the leakiness of envelope induced by ROS was further confirmed by determining ion leakage. As seen in Figure 5H, control (-PQ and -light) also showed ion leakage as a consequence of leaf disc preparation. However, the ion leakage increased much further when more stress (light and PQ) was applied. This could be explained by the fact that paraquat acts on PSI and in the light condition, excess ROS is generated. Paraquat (PSI inhibitor) intercepts the electrons destined for ferredoxin and NADP reduction and then reduces oxygen to superoxide in the light. This free radical reacts nonspecifically with a wide range of molecules in the chloroplast, leading to lipid peroxidation and disruption of chlorophyll. This compromises integrity of cell membranes and the cells as well as increases leakiness of organelles [58], [59]. Hence, the increased ion leakage in the presence of paraquat and light is due to increased ROS generated in the chloroplast, which in turn increases lipid peroxidation and decreases membrane integrity.

In both tobacco and lettuce chloroplasts after infection of leaf discs with *E. carotovora*, GFP moved towards the chloroplast envelope and was released into the cytoplasm from intact chloroplasts as evidenced by thylakoid integrity, chlorophyll fluorescence and chloroplast envelope. The same process of GFP release was observed in the transplastomic plants expressing PTD-GFP or GFP-RC101 (antimicrobial peptide) inoculated with *E. carotovora* but at a slower pace of infection (24–48 hr). While control PTD-GFP plants succumbed to the infection, GFP-RC101 plants showed enhanced resistance to infection up to 48 hrs, by releasing GFP-RC101 outside chloroplasts to kill *E. carotovora* (10^9^ PTD-GFP vs 10^4^ in GFP-RC101) so that Erwinia-caused pathological symptom was blocked, which eventually made the GFP-RC101 plants retain their overall photosynthetic efficiency (Figure 9E). This provides further evidence that the release is not due to the lysis of chloroplasts but could indeed be a tightly regulated process that requires further in depth investigation. Furthermore, PTD or any other protein transduction domain is not required for such release but it could be caused by the leakage of chloroplast envelope. Small spherical bodies were also detected (Figure 8A and 8B) which could act as retrograde signals, taking proteins out of the chloroplast into the cytoplasm. After concanamycin A treatment, spherical bodies have been detected in roots and excised leaves [60], [61].

ROS produced by host plants under pathogen attack can be used for the establishment of defenses such as hypersensitive response [62]. To cope with the defense line built by the host, plant-associated bacteria have also evolved tightly regulated, complex and specific oxidative stress responses against ROS produced by their hosts to protect themselves [63]. In the present study, ROS was unable to protect Erwinia infection because PTD transplastomic plants showed the severity of infection whereas GFP-RC101 plants were protected from Erwinia infection and fully recovered within 48 hr. This observation shows that along with the ROS signaling, the RC101 protein released from chloroplasts conferred protection against Erwinia infection and the confocal microscope images confirm the release of GFP fused with RC101.

In conclusion, these investigations provide direct evidence for release of GFP from chloroplasts regulated by ROS. It is likely that regulatory proteins are released from chloroplasts in response to stress and that protein trafficking is not unidirectional. These new concepts should help further understand hitherto unknown mechanism of retrograde signaling, especially the role of chloroplast proteins regulating nuclear genes, and offer new opportunities for chloroplast genetic engineering to regulate pathways outside this cellular compartment.
